# Prospective Detection of Early Lung Cancer in Patients With COPD in Regular Care by Electronic Nose Analysis of Exhaled Breath

**DOI:** 10.1016/j.chest.2023.04.050

**Published:** 2023-05-19

**Authors:** Rianne de Vries, Niloufar Farzan, Timon Fabius, Frans H.C. De Jongh, Patrick M.C. Jak, Eric G. Haarman, Erik Snoey, Johannes C.C.M. In ’T Veen, Yennece W.F. Dagelet, Anke-Hilse Maitland-Van Der Zee, Annelies Lucas, Michel M. Van Den Heuvel, Marguerite Wolf-Lansdorf, Mirte Muller, Paul Baas, Peter J. Sterk

**Affiliations:** a Amsterdam University Medical Centers, University of Amsterdam, Amsterdam University Medical Centers, University of Amsterdam, Amsterdam, The Netherlands; b Emma Children’s Hospital, Amsterdam University Medical Centers, University of Amsterdam, Amsterdam, The Netherlands; c The Netherlands Cancer Institute, Amsterdam, The Netherlands; d Breathomix B.V, Leiden, The Netherlands; e Medisch Spectrum Twente, Enschede, The Netherlands; f Franciscus Gasthuis & Vlietland, Rotterdam, The Netherlands; g Diagnostiek voor U, Eindhoven, The Netherlands; h Radboud University Medical Center, Nijmegen, The Netherlands

**Keywords:** breath test, breathomics, COPD, early detection, eNose, lung cancer

## Abstract

**Background:**

Patients with COPD are at high risk of lung cancer developing, but no validated predictive biomarkers have been reported to identify these patients. Molecular profiling of exhaled breath by electronic nose (eNose) technology may qualify for early detection of lung cancer in patients with COPD.

**Research Question:**

Can eNose technology be used for prospective detection of early lung cancer in patients with COPD?

**Study Design and Methods:**

BreathCloud is a real-world multicenter prospective follow-up study using diagnostic and monitoring visits in day-to-day clinical care of patients with a standardized diagnosis of asthma, COPD, or lung cancer. Breath profiles were collected at inclusion in duplicate by a metal-oxide semiconductor eNose positioned at the rear end of a pneumotachograph (SpiroNose; Breathomix). All patients with COPD were managed according to standard clinical care, and the incidence of clinically diagnosed lung cancer was prospectively monitored for 2 years. Data analysis involved advanced signal processing, ambient air correction, and statistics based on principal component (PC) analysis, linear discriminant analysis, and receiver operating characteristic analysis.

**Results:**

Exhaled breath data from 682 patients with COPD and 211 patients with lung cancer were available. Thirty-seven patients with COPD (5.4%) demonstrated clinically manifest lung cancer within 2 years after inclusion. Principal components 1, 2, and 3 were significantly different between patients with COPD and those with lung cancer in both training and validation sets with areas under the receiver operating characteristic curve of 0.89 (95% CI, 0.83-0.95) and 0.86 (95% CI, 0.81-0.89). The same three PCs showed significant differences (*P* < .01) at baseline between patients with COPD who did and did not subsequently demonstrate lung cancer within 2 years, with a cross-validation value of 87% and an area under the receiver operating characteristic curve of 0.90 (95% CI, 0.84-0.95).

**Interpretation:**

Exhaled breath analysis by eNose identified patients with COPD in whom lung cancer became clinically manifest within 2 years after inclusion. These results show that eNose assessment may detect early stages of lung cancer in patients with COPD.

**Take-home Points**

**Study Question:** Can electronic nose (eNose) technology be used for prospective detection of early lung cancer in patients with COPD?

**Results:** The eNose was able to discriminate patients with COPD who subsequently received a lung cancer diagnosis from those who did not receive such a diagnosis with 87% accuracy, an area under the receiver operating characteristic curve of 0.90 (95% CI, 0.84-0.95), 86% sensitivity, and 89% specificity.

**Interpretation:** These results show that eNose assessment may detect early stages of lung cancer in patients with COPD and therefore may be of value in screening this risk group.

Patients with COPD are at higher risk of lung cancer developing, with studies showing a relative risk of twofold to fourfold compared with the general population.^1^ Although several biomarkers are candidates for lung cancer discovery, such as autoantibodies, complement fragments, microRNA, circulating DNA, DNA methylation, RNA and protein profiling, and metabolomics,^2^ no validated biomarkers have been discovered that can identify patients with COPD who are at a higher risk of lung cancer developing.

During the past decade, screening studies using low-dose CT imaging have shown significant reductions (up to 39% in women) in lung cancer mortality rates among high-risk individuals, who were defined based on tobacco use history (status, pack-years, quit time) and age (50-75 years).^3^, ^4^, ^5^ Interestingly, a recently published study showed that patients with COPD are at higher risk of lung cancer regardless of tobacco use history.^6^ Because the potential benefits of screening might be exceeded by the increased risk of death inherent to COPD and its associated comorbidities, concerns exist about the inclusion of these patients in lung cancer screening programs.^7^^,^^8^ In addition, concerns regarding increased overdiagnosis by low-dose CT imaging and invasive follow-up investigations limit its applicability to patients with COPD.^9^ Therefore, an urgent need exists for an accurate and noninvasive test that can be implemented at the point of care. Specifically, a test that can refine the selection procedure of high-risk individuals for further follow-up screening tests is likely to prevail in clinical practice.

Molecular profiling of exhaled breath by electronic nose (eNose) technology may qualify for the early detection of lung cancer.^2^ eNose technology is an appealing noninvasive approach that applies advanced pattern recognition algorithms for analysis of the mixture of volatile organic compounds (VOCs) in exhaled breath.^10^^,^^11^ The thousands of VOCs present in exhaled breath reflect the metabolic processes occurring in the host both locally in the airways and systemically.^12^ Comprehensive analysis of these VOC patterns (breathomics) provides opportunities for noninvasive biomarker discovery in lung cancer.^10^^,^^13^^,^^14^ By using eNoses, differences in exhaled VOC patterns of individuals with COPD, individuals with lung cancer, and healthy individuals already have been demonstrated by multiple research groups.^11^^,^^15^, ^16^, ^17^, ^18^, ^19^ Therefore, we hypothesized that metabolic and molecular changes that occur in early stage asymptomatic lung cancer can be detected from exhaled breath using an eNose.

This study aimed to determine the diagnostic accuracy of exhaled breath analysis by eNose for (1) the discrimination between patients with COPD and those with lung cancer in a training and validation set and (2) the prospective prediction of early lung cancer in COPD. By using this stepwise approach and transparent reporting, the study follows the recommendations of the Standards for Reporting of Diagnostic Accuracy Studies guidelines^20^ and the Transparent Reporting of a Multivariable Prediction Model for Individual Prognosis or Diagnosis statement.^21^

## Study Design and Methods

### Study Population

BreathCloud is a real-world multicenter observational study in healthy control participants and participants with a suspected or established diagnosis of asthma, COPD, or lung cancer.^22^ All patients who visited the lung function departments for diagnostic and monitoring assessments in day-to-day care were recruited sequentially. The presently reported data include results from 682 patients with COPD and 211 patients with lung cancer who were included between May 2017 and November 2018. Patients with COPD were characterized according to the Global Initiative for Chronic Obstructive Lung Disease criteria^23^ and an established medical diagnosis of lung cancer was based on current guidelines.^24^^,^^25^ Exclusion criteria for participating in this study were the recent (< 12 h) intake of alcohol or if patients were not willing or able to participate. No further restrictions (eg, eating, drinking, tobacco use, or medication use) on participation were made to increase the applicability of breath analysis by eNose in clinical practice.

The ethics board of all participating centers concluded in writing that Dutch legislation on human participation in research was not considered to be applicable, given the noninvasive and minimally bothering nature of this study that merely added exhaled breath analysis by eNose to standard diagnostic procedures.^22^^,^^26^^,^^27^ Despite the waiver that was provided by the ethics review board, the purpose of adding the eNose to routine diagnostics was explained to the patients, all of whom gave their oral consent.

### Study Design and Measurements

The study had an observational prospective follow-up design. At baseline all patients with COPD underwent eNose assessment. The patients with COPD were treated according to standard care, and the subsequent incidence of clinically diagnosed lung cancer was assessed prospectively based on documented clinical records by following up the patients prospectively for 2 years. An established medical diagnosis of lung cancer was confirmed with CT scan imaging and was based on current guidelines.^24^^,^^25^

Clinical assessment of patients with COPD was performed using the Clinical COPD Questionnaire.^28^ In addition, the personal best FEV_1_ % predicted after bronchodilation was used from data collected up to 12 months before inclusion. Other clinical data were collected for routine clinical care and subsequently were handled by complying with the Wet Bescherming Persoonsgegevens (Dutch Personal Data Protection Act).

### Exhaled Breath Analysis

**Figure 1:**
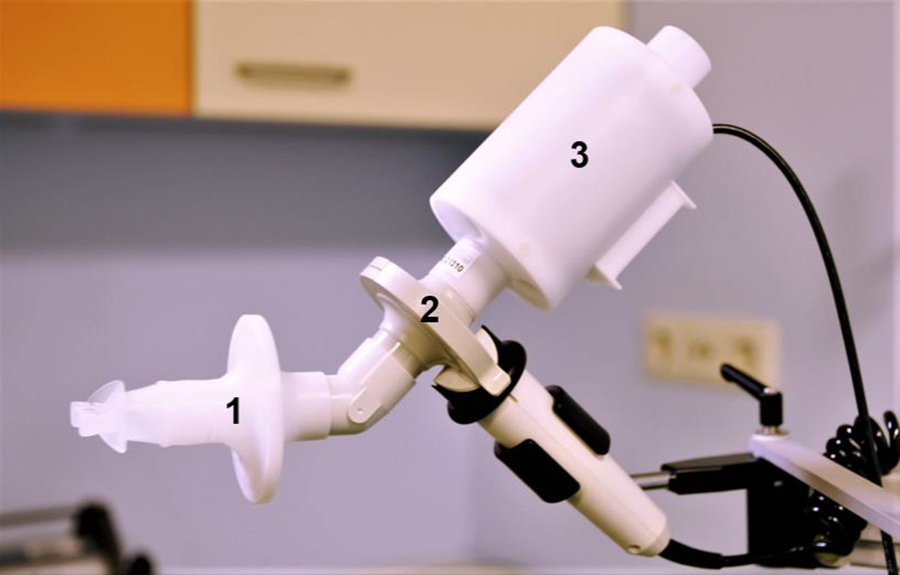
Photograph showing the SpiroNose measurement setup: (1) mouthpiece, nose clamp, and bacteria filter; (2) spirometer; and (3) SpiroNose.

Exhaled breath measurements were performed in duplicate using a technically and clinically validated eNose, the SpiroNose.^22^^,^^26^^,^^27^^,^^29^ The SpiroNose consists of seven different cross-reactive metal-oxide semiconductor sensors (sensors 1-7) for sampling of exhaled air. Another set of the same sensors sampled ambient air for background correction (Fig 1). SpiroNose measurements comprise five tidal breaths followed by an inspiratory capacity maneuver to total lung capacity and a 5-s breath hold followed by a slow expiration (< 0.4 L/s) to residual volume. The raw SpiroNose sensor signals were sent in real time to an online analysis platform for automated data analysis. An in-depth description of the measurement setup and the verification of the sensor stability is published elsewhere.^22^^,^^26^

### Data Processing

The processing of the eNose sensor deflections was carried out automatically using the standard eNose software as was published previously.^22^^,^^27^ Signal processing included signal detrending, filtering, ambient air correction, automatic peak detection, and parameter selection. From each sensor signal, two variables were determined: (1) the highest sensor peak normalized to the most stable sensor, sensor 2, to minimize inter array differences and (2) the ratio between the sensor peak and the breath hold point.^22^^,^^26^ The sensor peak ratios and peak to breath hold ratios were used for statistical analysis.

### Statistical Analysis

A principal component analysis (PCA) was performed to merge the variables of interest into a multivariate component. According to the Kaiser criterion, all principal components (PCs) with an eigenvalue of > 1 were retained.^30^ The processed sensor variables, the original sensor peaks, and the peak to breath hold ratios were restructured to four PCs that captured 78.4% of the variance within the dataset (PC 1, 39.8%; PC 2, 19.5%; PC 3, 11.1%; and PC 4, 8.0%). PCs were constructed for all participants (training and validation set) based on eNose data from participants within the training set (e-Table 1). Reducing the dimensionality of the data before machine learning is preferred to reduce the risk of overfitting.^31^^,^^32^

### COPD vs Lung Cancer

Analyses were performed in both a training and validation set defined by random split analysis (2:1), as recommended for metabolomics experiments.^33^ The obtained PCs were compared between groups using independent *t* tests. The PCs that discriminated (*P* < .05) between groups were selected for further analysis. The *t* tests were validated internally by 1,000 bootstrap iterations. Subsequently, linear discriminant analysis was performed using the selected variables. The aim of linear discriminant analysis is to maximize the separability of the defined categories and represents a relatively simple and portable algorithm. The latter is recommended for eNose research^31^ and often is used in clinical studies performing exhaled breath analysis with the SpiroNose.^26^^,^^27^ Based on the differentiating PCs, a discriminant function was calculated that best distinguished between groups. The accuracy of this model was defined as the percentage of correctly classified patients in the training set. Leave-one-out cross-validation was used to calculate the cross-validated accuracy value (percentage). The discriminant scores were used to construct a receiver operating characteristic curve, including the area under the receiver operating characteristic curve (AUC) and corresponding 95% CI. Finally, the discriminant function obtained from the training set was examined in the independent validation set and compared mutually based on AUC curves.

As a first step in the assessment of early lung cancer, the training and validation sets were combined and the accuracy, sensitivity, and specificity for the discrimination between COPD and lung cancer were assessed across the entire study population. A PCA plot was created to visualize the data. Finally, the influence of comorbid COPD in the lung cancer group on the accuracy of distinguishing lung cancer from COPD was assessed by removing all patients with a double diagnosis from the analysis (e-Appendix 1, e-Fig 1, e-Table 2).

### Other Classification Algorithms

To avoid drawing conclusions based on a single classification model, we used three additional and powerful machine learning techniques to classify the eNose data: gradient boosting machine, adaptive least absolute shrinkage and selection operator, and sparse partial least squares discriminant analysis. These machine learning techniques all have been used before in metabolomics research^34^, ^35^, ^36^ and provide an estimate of the robustness of the statistical performance across different models (e-Appendix 1, e-Tables 3, 4). To contribute to the standardization of the SpiroNose’s methods, we chose to present the linear discriminant analysis results throughout herein.

### Prospective Early Lung Cancer Detection

Similar analyses including PCA, independent *t* tests, linear discriminant analysis, and receiver operating characteristic curve analysis were used to determine the diagnostic accuracy of eNose analysis for the discrimination between patients with COPD who did and did not receive a clinical diagnosis of lung cancer within 2 years after inclusion. In addition, sensitivity, specificity, a positive likelihood ratio (LR), and a negative LR were calculated. A PCA plot was created to visualize the data. A duplicate of this plot was created, in which the COPD group that did receive a clinical diagnosis of lung cancer within 2 years was divided into the different lung cancer stages (e-Fig 2).

### Clinical and Biological Determinants on the eNose Data

A multiple linear regression was calculated to predict eNose data based on the clinical metadata. More information on the analysis and results is presented in e-Tables 5-7.

## Results

### COPD vs Lung Cancer

In total, 893 patients (682 patients with COPD and 211 patients with lung cancer) were included in this study, of whom 596 patients (455 patients with COPD and 141 patients with lung cancer) were included for training of the results and 297 patients (227 patients with COPD and 70 patients with lung cancer) for validation of the results (Table 1). In the training set, only pack-years of tobacco use and FEV_1_ showed significant differences (*P* < .05) between patients with COPD and those with lung cancer. No significant differences were found between these groups in baseline characteristics in the validation set. In addition, the baseline characteristics of patients in the training and validation set were similar, except for significantly different COPD staging (*P* < .05) and lung cancer pathologic features (*P* < .05) (Table 1).

**Table 1:** Baseline Characteristics of Patients With COPD and Those With Lung Cancer From Both the Training and Validation Sets

Variable	Training Set		Validation Set	
	COPD (n = 455)	Lung Cancer (n = 141)	COPD (n = 227)	Lung Cancer (n = 70)
Male sex	191 (42)	75 (53)	91 (40)	33 (47)
Age, y	64.3 ± 9.4	63.0 ± 9.9	63.1 ± 8.1	64.6 ± 10.9
BMI, kg/m^2^	26.2 ± 5.4	25.2 ± 4.8	27.9 ± 6.8	26.1 ± 4.4
Tobacco use history				
Never	3 (0)	14 (10)	1 (0)	7 (10)
Former	334 (73)	92 (65)	174 (77)	48 (69)
Current	118 (26)	35 (25)	52 (23)	15 (21)
Pack-y of tobacco use	37.6 (31.1-55.8)^a^	29.4 (25.9-47.3)^a^	39.1 (31.9-53.8)	35.2 (29.3-45.3)
White race	398 (87)	127 (90)	205 (90)	22 (97)
FEV_1_, % predicted	63.7 ± 18.4^a^	72.0 ± 20.1^a^	68.7 ± 23.5	69.4 ± 18.9
COPD staging, GOLD classification	455 (100)	82 (58)	227 (100)	34 (49)
I	41 (9)	20 (14)	18 (8)	5 (7)
II	245 (54)^b^	39 (28)	93 (41)^b^	23 (32)
III	129 (28)^b^	22 (16)	89 (39)^b^	6 (9)
IV	40 (9)	1 (0)	27 (12)	0 (0)
Pathologic findings				
SCLC	NA	17 (12)	NA	11 (16)
NSCLC	NA	124 (88)	NA	59 (84)
Adenocarcinoma	NA	87 (62)	NA	35 (50)
Squamous cell carcinoma	NA	22 (16)^b^	NA	18 (26)^b^
Large cell carcinoma	NA	15 (11)	NA	6 (9)
Stage				
I	NA	17	NA	5
II	NA	31	NA	16
III	NA	48	NA	29
IV	NA	45	NA	20

Data are presented as No. (%), No., mean ± SD, or median (interquartile range). GOLD = Global Initiative for Chronic Obstructive Lung Disease; NA = not applicable; NSCLC = non-small cell lung cancer; SCLC = small cell lung cancer.

a Significant difference between COPD and lung cancer group (*P* < .05).

b Significant difference between training and validation set (*P* < .05).

In the training set, PC 1 (*P* = .039), PC 2 (*P* < .001), and PC 3 (*P* < .001) were significantly different between patients with COPD and those with lung cancer. Subsequent discriminant analysis showed a cross-validated accuracy value of 88%. The AUC ± 95% CI after internal cross-validation reached 0.89 (95% CI, 0.83-0.95). The ability to discriminate between COPD and lung cancer, with PC 1, PC 2, and PC 3 as input for the model obtained from the training set, was confirmed in an independent validation set. Breathprints of patients with COPD and those with lung cancer in the validation set were distinguished with a cross-validated accuracy value of 83% and an AUC of 0.86 (95% CI, 0.81-0.89). The results of the other classification algorithms in the validation set were similar and are presented in e-Tables 3 and 4.

**Figure 2:**
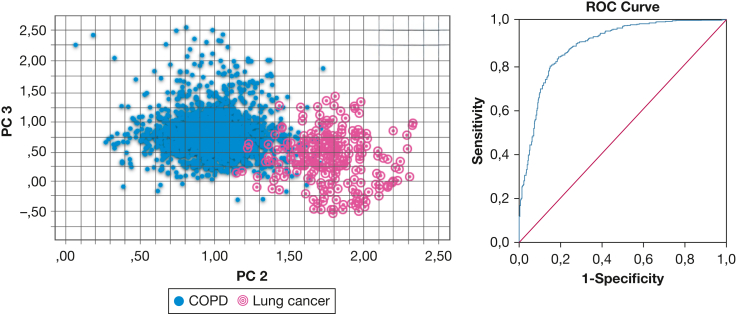
A, Two-dimensional plot showing the discrimination of breath profiles between patients with COPD and those with lung cancer (combined training and validation set) along PC 2 and PC 3 (PCs with the highest discriminative value). B, Graph showing the ROC curve with line of identity for the identification of lung cancer of 0.86 (95% CI, 0.81-0.89). PC = principal component; ROC = receiver operating characteristic.

For completeness and as a first step toward assessment of early lung cancer, we combined both the training and validation sets. PC 2 (*P* < .001) and PC 3 (*P* < .001) were significantly different between COPD and lung cancer. Groups were distinguished with a cross-validated accuracy of 84% and the AUC reached 0.86 (95% CI, 0.82-0.94) (Fig 2). When excluding all patients with lung cancer with comorbid COPD from the analysis (e-Table 2, e-Fig 1), a cross-validated accuracy of 90% and AUC of 0.95 (95% CI, 0.92-0.97) were reached.

### Prospective Early Lung Cancer Detection

**Figure 3:**
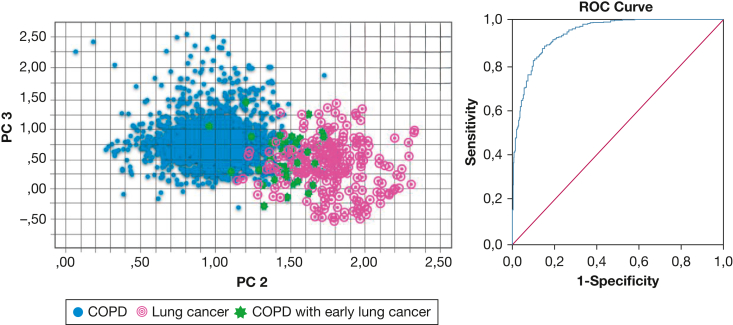
A, Two-dimensional plot showing the discrimination of breath profiles between patients with COPD and those with COPD who demonstrated clinically manifest lung cancer within 2 years after inclusion in the study (COPD with early lung cancer) along PC 2 and PC 3. The lung cancer breath profiles are plotted as a reference. B, Graph showing ROC curve with line of identity for the identification of patients with COPD with early lung cancer of 0.90 (95% CI, 0.84-0.95). PC = principal component; ROC = receiver operating characteristic.

Of the total 682 patients with COPD, 37 patients (5.4%) demonstrated clinically manifest lung cancer within 2 years after inclusion (Table 2). PC 1 (*P* = .002), PC 2 (*P* < .001), and PC 3 (*P* < .001) showed a significant difference at baseline between the patients with COPD who did and did not receive a diagnosis of lung cancer within 2 years, with a cross-validated accuracy value of 87%. The AUC after internal cross-validation reached 0.90 (95% CI, 0.84-0.95). Thirty-three of these 37 patients with COPD (89%) were classified by eNose as having lung cancer already at baseline (Fig 3). The identification of patients with COPD who demonstrated clinically manifest lung cancer resulted in 86% sensitivity, 89% specificity, a positive LR of 7.80, and a negative LR of 0.15.

**Table 2:** Baseline Characteristics of Patients With COPD Who Did and Did Not Demonstrate Clinically Manifest Lung Cancer Within 2 Years After Inclusion

Variable	Clinically Manifest Lung Cancer Did Not Develop (n = 645)	Clinically Manifest Lung Cancer Did Develop (n = 37)
Male sex	268 (42)	14 (38)
Age, y	63.7 ± 14.2	64.5 ± 16.9
BMI, kg/m^2^	26.9 ± 5.0	25.7 ± 6.2
Tobacco use history		
Never	4 (0)	0 (0)
Former	483 (75)	25 (68)
Current	158 (24)	12 (32)
Pack-y of tobacco use	38.2 (31.2-55.4)	36.1 (30.3-53.0)
White race	572 (89)	31 (84)
FEV_1_, % predicted	65.0 ± 20.1	69.4 ± 18.9
COPD staging, GOLD classification		
I	59 (9)	0 (0)
II	327 (51)^a^	11 (30)^a^
III	202 (31)	16 (43)
IV	57 (9)^a^	10 (27)^a^
Pathologic findings		
SCLC	NA	5 (14)
NSCLC	NA	32 (86)
Adenocarcinoma	NA	17 (46)
Squamous cell carcinoma	NA	14 (38)
Large cell carcinoma	NA	1 (3)
Staging		
I	NA	5
II	NA	12
III	NA	13
IV	NA	7

Data are presented as No. (%), No., mean ± SD, or median (interquartile range). GOLD = Global Initiative for Chronic Obstructive Lung Disease; NA = not applicable; NSCLC = non-small cell lung cancer; SCLC = small cell lung cancer.

a Significant difference between COPD and early lung cancer group (*P* < .05).

### Lung Cancer Stage

The COPD group that did receive a clinical diagnosis of lung cancer was divided into early and advanced stage lung cancer (e-Fig 2). PC 3 (*P* < .001) was significantly different between early (stages I and II) and advanced (stages III and IV) lung cancer. Stages were distinguished with a cross-validated accuracy of 88% and an AUC that reached 0.93 (95% CI, 0.87-0.98).

## Discussion

This study showed that exhaled breath analysis using eNose is able to distinguish between lung cancer and COPD. These results were confirmed in an independent validation set. Additionally, of the 37 patients with COPD who received a clinical diagnosis of lung cancer within 2 years after inclusion in the study, 33 were classified correctly as having lung cancer by eNose already at baseline. The eNose was able to discriminate patients with COPD who subsequently received a lung cancer diagnosis from those who did not with 87% accuracy.

To our knowledge, this is the first prospective discovery study of early lung cancer among patients with COPD by using eNose. Distinct exhaled VOC patterns of lung cancer vs healthy individuals long have been demonstrated by researchers worldwide, including favorable accuracies using various eNose devices.^11^^,^^15^, ^16^, ^17^, ^18^, ^19^^,^^37^^,^^38^ In the past few years, however, several research groups have taken further steps to assess the potential of exhaled breath analysis for discrimination between lung cancer and COPD, two closely associated diseases. The present study showed accurate discrimination of lung cancer and COPD, and more so when double diagnoses were excluded. Dragonieri et al^15^ also showed significant differences in breath profiles of participants with non-small cell lung cancer, participants with COPD, and healthy control participants in a small eNose study. Similar to our results, they were able to distinguish between the groups with 85% accuracy. Another study using gas chromatography-mass spectrometry showed that levels of several VOCs, including isoprene and methyl pentane, were significantly higher in non-small cell lung cancer compared with COPD.^17^ Increased levels of isoprene in the breath of patients with lung cancer have been reported in numerous studies.^39^, ^40^, ^41^ One of the important conclusions of the study by Poli et al^17^ is that none of the VOCs alone was able to distinguish between the two groups adequately. In fact, the highest accuracy (83%; with 72% sensitivity and 94% specificity) was achieved when combining 13 VOCs. Indeed, considering the complexity of the two diseases regarding their underlying cellular and molecular pathways, difficulties in identifying highly informative single biomarkers are not surprising. Therefore, technologies such as eNose that capture the complete mixture of VOCs combined with pattern recognition algorithms are highly valuable in identifying composite biomarkers, providing better insights into the actual condition of patients.^11^

In the current study, we used a technically and clinically validated eNose linked to an online cloud solution to perform exhaled breath measurements.^22^^,^^26^ It should be noted that eNoses can differ significantly in number and type of sensors as well as algorithms used for signal processing, statistical analysis, or both.^42^ In a study using eNose technology, 11 of 23 patients with COPD (47%) were identified as having lung cancer and only one of those patients received a clinical diagnosis of lung cancer within 56 months.^16^ As concluded by the authors, a lack of sufficient sample size for adequate training of the eNose could be one of the reasons for suboptimal distinction. The present study, using a bigger sample size, distinct signal processing, and dissimilar statistical analysis using in total four classification models, seemed to confirm this.

The present study was a spin-out of the multicenter BreathCloud project. A potential bias could be that patients with COPD were recruited mostly from secondary care centers and had received a diagnosis clinically according to the Global Initiative for Chronic Obstructive Lung Disease criteria. In the Netherlands, patients with mild to moderate COPD are managed mainly at primary care centers by general practitioners, whereas patients with more severe forms of the disease are managed at secondary care centers. The presence of moderate or severe COPD is shown to be associated with a higher risk of lung cancer,^6^ which could explain the relatively high lung cancer incidence in the present study population (5%) compared with that of previous reports (approximately 1%).^43^^,^^44^

Tobacco use is known to influence the composition of exhaled VOCs, and therefore is considered to be a potential confounder in exhaled breath analysis.^45^ Although pack-years of tobacco use drove the prediction of PC 2 (not PC 1 or PC 3) significantly, tobacco use status did not. It could very well be that hematologic parameters are affected with long-term tobacco use and that this could affect exhaled breath profiles.^46^ Although the median pack-years of tobacco use in patients with COPD were higher than in patients with lung cancer in the training set, no significant differences were found between the two groups in the validation set. For this reason and because pack-years of tobacco use did not contribute significantly to the prediction of PC 1 or PC 3, tobacco use is unlikely to have influenced our findings, consistent with a previous report.^47^ Furthermore, our analysis demonstrated no significant differences regarding tobacco use status or pack-years of tobacco use in patients with COPD who received a lung cancer diagnosis compared with those who did not.

Furthermore, age, BMI, ethnicity, lung cancer staging, and lung cancer pathologic features also added significantly to the prediction of one or two PCs (eNose data). However, these baseline characteristics in the training and validation set were similar for patients with COPD and those with lung cancer and were similar between the training and validation set, except for lung cancer pathologic features. The validation set contained slightly more patients with squamous cell carcinoma, which may explain in part the small difference in results between the training and validation set.

One of the main strengths of our study is its prospective, real-world design and the use of an independent validation set and multiple classification algorithms for distinguishing between lung cancer and COPD that confirmed the results of the training set. Another strength of the study is its use of a technically validated eNose. The extensive technical validation of the eNose was performed regarding the development of signal processing, ambient air correction, and data analysis techniques as well as identifying the optimal measurement maneuver (eg, flow, volume) and influence of environmental factors (eg, humidity, temperature) on breath measurements.^27^ Our study certainly also has limitations. The real-world design lacked CT scan imaging at baseline. Therefore, it is unclear whether a tumor already was present at baseline. None of the patients with COPD showed symptoms suggestive of lung cancer at that time. Still, it cannot be excluded that, for some patients with COPD with a subsequent diagnosis of lung cancer, a baseline CT scan might have detected the existing tumor. However, evidence that imaging techniques exhibit high false-positive rates is good, while also showing that these methods have difficulty in distinguishing between benign and malignant tumors.^3^^,^^48^, ^49^, ^50^

## Interpretation

The data suggest that cancer-related cellular and metabolic processes already were present at the time of exhaled breath measurement despite the absence of any symptoms suggestive of lung cancer. Therefore, the obtained breath profiles in these patients seem to reflect the composition of VOCs that arise from both COPD and lung cancer. Baseline breath profiles of patients with COPD who subsequently demonstrated clinically manifest lung cancer were comparable with those in whom COPD and lung cancer coexist. However, because eNoses analyze the complete mixture of VOCs, it remains to be elaborated whether VOCs arise from cancer cells, the host’s immunologic or inflammatory responses, or the tumor microbiome.^51^

Interestingly, the VOC pattern associated with early development of lung cancer in COPD did not match to the pattern related to lung cancer stages: the former was mainly captured by PC 2 and the latter by PC 3 (e-Fig 2). This suggests that early identification of upcoming clinically manifest lung cancer in patients with COPD by eNose analysis is not driven by VOCs that are associated predominantly with a particular stage of the disease.

What could be the implications of this study? According to the Global Initiative for Chronic Obstructive Lung Disease report, lung cancer is the main cause of death among patients with COPD. The eligibility criteria of current lung cancer screening programs are limited to age and tobacco use history, and they show high false-positive rates.^3^ It is becoming more evident that COPD is a risk factor for lung cancer. Recently, the HUNCHEST screening program reported higher relative risk for malignant tumor entity in patients with COPD compared with patients without COPD (relative risk, 1.85; 95% CI, 0.85-4.07).^52^ Moreover, COPD has been shown to be an independent risk factor for lung cancer regardless of the tobacco use status of the patient because a large-scale study demonstrated that those with COPD who have never used tobacco have a 2.6-times higher risk of lung cancer developing compared with those without COPD who have never used tobacco.^6^ Therefore, a large number of lung cancer cases may not be detected because of the exclusion of current and former tobacco users with COPD from screening programs,^6^^,^^53^ whereas the number of missed cases might be even higher when considering patients with COPD who do not use tobacco. Therefore, optimizing the screening criteria for lung cancer by including patients with COPD seems to be recommended. Early detection not only results in potentially curative intervention, but it also can decrease the risk of life-threatening complications of treatment. Our data suggest that eNose technology can capture lung cancer-specific VOC patterns in its early development, which provides a window for potentially curable interventions.

In conclusion, exhaled breath analysis by eNose examination identified patients with COPD in whom lung cancer subsequently became clinically manifested within 2 years after inclusion. These results show that eNose assessment may provide a novel means for early identification of patients with COPD at risk of malignancy and may improve patient outcomes by identifying those who will benefit most from further diagnostic procedures and early intervention.

## Funding/Support

The BreathCloud project was sponsored by the Lung Foundation Netherlands and the Dutch VriendenLoterij. The study described in this article is a spin-out of the BreathCloud project and was carried out without additional funding.

## Financial/Nonfinancial Disclosures

The authors have reported to *CHEST* the following: R. d. V. receives personal fees and has a substantial interest in the start-up company Breathomix BV. N. F. and Y. W. F. D. receive personal fees from the start-up company Breathomix BV. P. J. S. is scientific adviser and has an officially nonsubstantial interest in the start-up company Breathomix BV. None declared (T. F., F. H. C. D. J., P. M. C. J., E. G. H., E. S., J. C. C. M. I. T. V., A.-H. M.-V. D. Z., A. L., M. M. V. D. H., M. W.-L., M. M., P. B.).

## Supplementary Data

e-Online Data
