# Supplementary material for: Prospective Detection of Early Lung Cancer in Patients With COPD in Regular Care by Electronic Nose Analysis of Exhaled Breath
Source: Chest. 2023 May 19;164(5):1315–24. doi: 10.1016/j.chest.2023.04.050 (PMC10635840; doi:10.1016/j.chest.2023.04.050)
Supplement: e-Online Data [file mmc1.docx]

**Supplementary material**

**Results**

A principal component analysis (PCA) was performed to merge the variables of interest into a multivariate component. According to the Kaiser criterion, all PCs with an eigenvalue >1 were retained. The processed sensor variables; the original sensor peaks and the peak/breath-hold ratios were restructured to four PCs that captured 78.4% of the variance within the dataset (PC1 39.8%, PC2 19.5%, PC3 11.1% and PC4 8.0%). PCs were constructed for all subjects (training and validation set), based on eNose data from subjects within the training set (e-Table 1).

| **e-Table 1.** Component Matrix | | | | |
| --- | --- | --- | --- | --- |
|  | Component | | | |
|  | 1 | 2 | 3 | 4 |
| S1 | -.241 | .022 | .819 | .331 |
| S3 | .327 | .514 | .100 | -.146 |
| S4 | .228 | .665 | .249 | .032 |
| S5 | .472 | .620 | -.042 | .153 |
| S6 | .133 | .726 | -.314 | .141 |
| S7 | .364 | -.358 | -.139 | .585 |
| S1BH | .782 | -.173 | -.104 | -.087 |
| S2BH | .788 | -.059 | -.113 | .146 |
| S3BH | .637 | -.255 | -.061 | .353 |
| S4BH | .830 | .003 | .174 | .036 |
| S5BH | .566 | -.169 | .410 | -.257 |
| S6BH | .759 | -.031 | .072 | -.084 |
| S7BH | .569 | -.197 | -.003 | -.484 |
| Extraction Method: Principal Component Analysis. | | | | |
|  | | | | |

**Influence of comorbid COPD**

The influence of comorbid COPD in the lung cancer group on the accuracy of distinguishing lung cancer from COPD was assed by removing all patients with a double diagnosis (n=116) from the analysis (e-Table 2). PC 1 (p=0.002), PC 2 (p<0.001) and PC 3 (p<0.001) showed a significant difference between COPD and lung cancer. When excluding all lung cancer patients with comorbid COPD from the analysis, a cross-validated accuracy of 90% and a ROC-AUC of 0.95 (CI: 0.92-0.97) was reached (e-Figure 1).


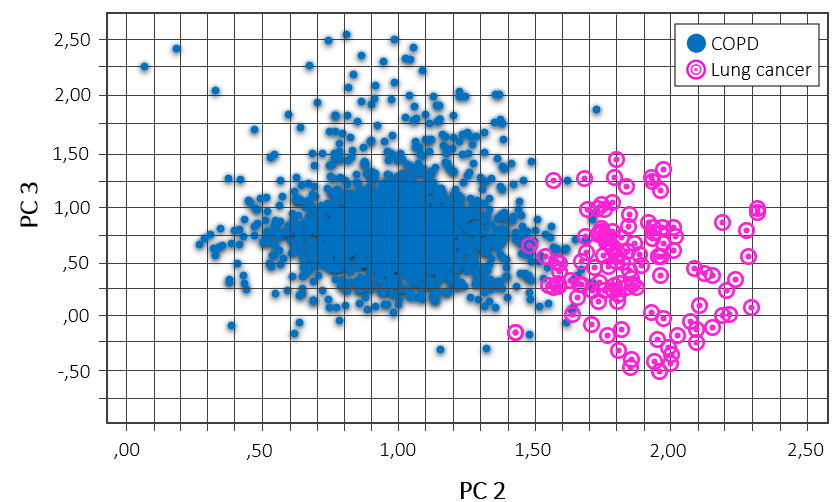

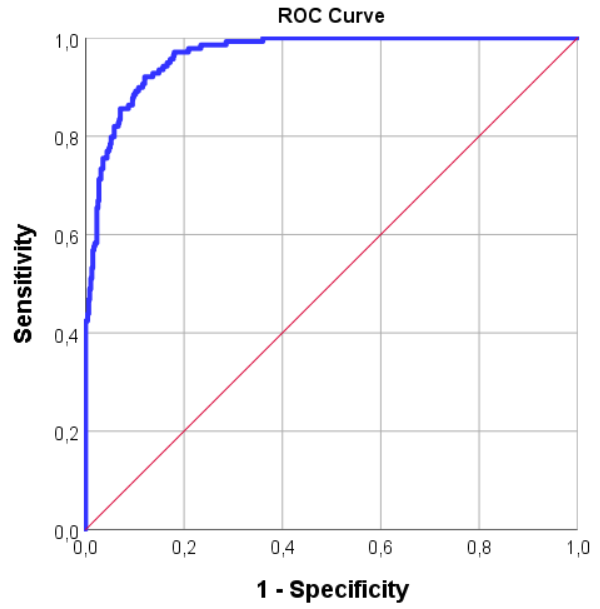


ROC-AUC: 0.95

**e-Figure 1.** (Left) Visualization of breath profiles of all COPD and lung cancer patients (combined training and validation set): Two-dimensional plot showing the discrimination of breath profiles between COPD and lung cancer patients (without comorbid COPD) along the principal components (PC2 and PC3) with the highest discriminative value. (Right) Receiver operating characteristic (ROC) curve with line of identity for the identification of lung cancer.

**e-Table 2.** Baseline characteristics of COPD and lung cancer patients without comorbid COPD of the combined training and validation set.

|  | **COPD**  **(n=682)** | **Lung cancer**  **(n=95)** |
| --- | --- | --- |
| Male sex – no.(%)  Age (yr) – mean (±SD)  BMI – mean (±SD)  Smoking (Never/Ex/Current)  Pack years – median (IQR)  Caucasian – no.(%)  FEV1 – mean (±SD)  *COPD staging – no. (%)*  GOLD I  GOLD II  GOLD III  GOLD IV  *Pathology – no. (%)*  SCLC  NSCLC  Adenocarcinoma  Squamous cell carcinoma  Large cell carcinoma  *Staging – no.*  Stage I/II/III/IV | 282 (41)  63.8 (14.4)  26.7 (5.1)  4/508/170  38.0 (23.9)  603 (88)  65.2 (19.9)^a^  59 (9)  338 (50)  218 (32)  67 (10) | 46 (48)  63.9 (9.0)  25.1 (5.3)  1/71/23  33.9 (19.9)  87 (92)  71.2 (18.5)^a^  12 (13)  83 (87)  52 (55)  24 (25)  7 (7)  9/34/32/20 |

^a^ Signiﬁcant difference between COPD and Lung cancer group (p<0.05); SD = Standard deviation; BMI = Body mass index;

IQR = interquartile range; FEV1 = Forced expiratory volume in 1 second; COPD = Chronic obstructive pulmonary disorder; GOLD = Global Initiative for chronic obstructive lung disease. SCLC = Small Cell Lung Cancer; NSCLC = Non-Small Cell Lung Cancer

**Other classification algorithms**

To avoid drawing conclusions based on a single classification model, we used three additional and powerful machine learning techniques to classify the eNose data: Gradient Boosting Machine (GBM), adaptive Least Absolute Shrinkage and Selection Operator (LASSO) and sparse Partial Least Squares Discriminant Analysis (sPLS-DA). These machine learning techniques have all been used before in metabolomics research [1-3] and provide an estimate of the robustness of the statistical performance across different models. Furthermore, these different models are used to evaluate the accuracy of exhaled breath analysis by eNose for the discrimination between COPD and lung cancer patients without potential bias from selecting a single model. The *training* set was used to train the additional 3 models based on the eNose data using internal cross-validation (e-Table 3). The predictive power of the fitted models was assessed in the independent *validation* set and compared based on ROC-AUC curves (e-Table 4).

**e-Table 3**. The ROC-AUCs with 95% CI for 4 different machine learning models on eNose data from the training set for the discrimination between COPD and lung cancer patients.

| **Methods** | **ROC-AUC** |
| --- | --- |
| GBM | 0.97 (CI:0.96-0.98) |
| Adaptive LASSO | 0.87 (CI:0.84-0.90) |
| sPLS-DA | 0.89 (CI:0.84-0.94) |
| LDA *(provided in manuscript)* | 0.89 (CI:0.83-0.95) |

GBM: Gradient Boosting Machine (GBM), Adaptive LASSO: adaptive Least Absolute Shrinkage and Selection Operator, sPLS-DA: sparse Partial Least Squares Discriminant Analysis, LDA: Linear Discriminant Analysis

**e-Table 4**. The ROC-AUCs with 95% CI for 4 different machine learning models on eNose data from the validation set for the discrimination between COPD and lung cancer patients.

| **Methods** | **ROC-AUC** |
| --- | --- |
| GBM | 0.88 (CI:0.80-0.94) |
| Adaptive LASSO | 0.86 (CI:0.83-0.89) |
| sPLS-DA | 0.87 (CI:0.81-0.91) |
| LDA *(provided in manuscript)* | 0.86 (CI:0.81-0.89) |

GBM: Gradient Boosting Machine (GBM), Adaptive LASSO: adaptive Least Absolute Shrinkage and Selection Operator, sPLS-DA: sparse Partial Least Squares Discriminant Analysis, LDA: Linear Discriminant Analysis

**Lung cancer staging**

The distribution of the different stages of lung cancer in the COPD group that developed clinically manifested lung cancer within 2 years after inclusion was investigated by splitting the group into the different lung cancer stages and plotting them on the COPD and lung cancer reference groups (e-Figure 2). PC 3 (p<0.001) showed a significant difference between early (stage I and II) and advance (stage III and IV) stage lung cancer. Lung cancer stages were distinguished with a cross-validated accuracy of 88% and the ROC-AUC reached 0.93 (CI:0.87-0.98).


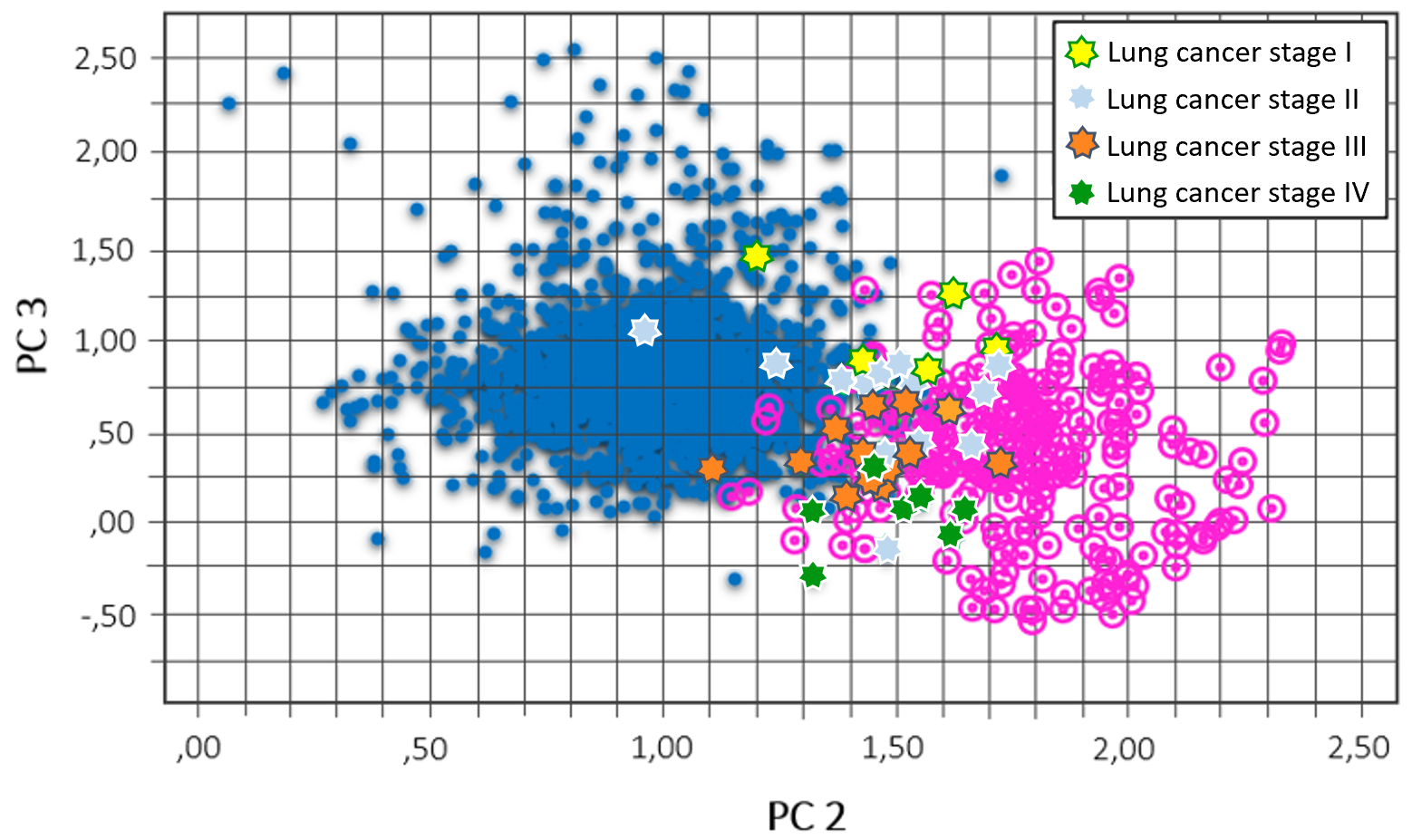


**e-Figure 2.** Visualization of breath profiles of all COPD and lung cancer patients (combined training and validation set): Two-dimensional plot showing the discrimination of breath profiles between COPD and lung cancer patients along the principal components (PC2 and PC3) with the highest discriminative value. The COPD group that did receive a clinical diagnosis of lung cancer within 2 years after inclusion is broken down into the different stages of lung cancer (stage I-IV).

**Multivariate regression analysis – clinical and biological determinants on the eNose data**

First, a multivariate linear regression, with stepwise selection of the independent variables, was calculated in the *training* set to predict eNose data (PC1, PC2 and PC3) from gender, age, BMI, ethnicity, lung function, smoking status, pack years, COPD/lung cancer staging and pathology.

The multiple regression model statistically significantly predicted PC1 (F(10,310)=5.715, p<0.001 with a R^2^ of 0.395), PC3 (F(10, 310)=1.983, p=0.035 with a R^2^ of 0.245) and PC2 (F(10, 310)=5.970, p<0.001 with a R^2^ of 0.402). Age, BMI, ethnicity, pack years, lung cancer staging, and pathology added significantly to the prediction of the eNose data, p<0.05. Regression coefficients and standard errors can be found in e-Tables 5-7.

**e-Table 5**. Summary of multiple regression analysis to predict PC1 in the training set

| Variable | B | SE_B_ | β |
| --- | --- | --- | --- |
| Intercept | -.262 | .747 |  |
| Gender | -.218 | .132 | -.102 |
| Age | .011 | .006 | .114* |
| BMI | .005 | .012 | .020 |
| FEV1 | .064 | .143 | .041 |
| Pack Years | .002 | .002 | .041 |
| Smoking status | -.125 | .094 | -.071 |
| Ethnicity | .074 | .011 | .371* |
| COPD staging | -.001 | .002 | -.018 |
| Lung cancer staging | -.003 | .002 | -.074 |
| Pathology | -.137 | .069 | -.177* |

* p<0.05; B = unstandardized regression coefficient; SEB = Standard error of the coefficient; β = standardized coefficient.

**e-Table 6**. Summary of multiple regression analysis to predict PC2 in the training set.

| Variable | B | SE_B_ | β |
| --- | --- | --- | --- |
| Intercept | -1.637 | .677 |  |
| Gender | -.107 | .120 | -.058 |
| Age | .009 | .005 | .105 |
| BMI | .024 | .011 | .126* |
| FEV1 | -.134 | .129 | -.101 |
| Pack Years | .004 | .002 | .118* |
| Smoking status | .078 | .085 | .052 |
| Ethnicity | .008 | .010 | .045 |
| COPD staging | .001 | .002 | .032 |
| Lung cancer staging | .001 | .002 | .038 |
| Pathology | .099 | .062 | .149 |

* p<0.05; B = unstandardized regression coefficient; SEB = Standard error of the coefficient; β = standardized coefficient.

**e-Table 7**. Summary of multiple regression analysis to predict PC3 in the training set

| Variable | B | SE_B_ | β |
| --- | --- | --- | --- |
| Intercept | .849 | .753 |  |
| Gender | .016 | .133 | .007 |
| Age | -.002 | .006 | -.019 |
| BMI | -.015 | .012 | -.065 |
| FEV1 | -.044 | .144 | -.028 |
| Pack Years | .003 | .002 | .065 |
| Smoking status | .018 | .095 | .010 |
| Ethnicity | -.075 | .011 | -.374* |
| COPD staging | -.001 | .003 | -.020 |
| Lung cancer staging | -.185 | .072 | -.266* |
| Pathology | .065 | .069 | .082 |

* p<0.05; B = unstandardized regression coefficient; SEB = Standard error of the coefficient; β = standardized coefficient.

**References**

1. Bouwmeester, R., L. Martens, and S. Degroeve, *Comprehensive and empirical evaluation of machine learning algorithms for small molecule LC retention time prediction.* Analytical chemistry, 2019. **91**(5): p. 3694-3703.

2. Cuperlovic-Culf, M., *Machine Learning Methods for Analysis of Metabolic Data and Metabolic Pathway Modeling.* Metabolites, 2018. **8**(1): p. 4.

3. Determan Jr, C.E., *Optimal algorithm for metabolomics classification and feature selection varies by dataset.* International journal of biology, 2015. **7**(1): p. 100.
